# AICAR activates the pluripotency transcriptional network in embryonic stem cells and induces KLF4 and KLF2 expression in fibroblasts

**DOI:** 10.1186/1471-2210-9-2

**Published:** 2009-02-12

**Authors:** Luigi Adamo, Yuzhi Zhang, Guillermo García-Cardeña

**Affiliations:** 1 Department of Pathology, Laboratory for Systems Biology, Center for Excellence in Vascular Biology, Harvard Medical School and Brigham and Women's Hospital, Boston (MA), USA

## Abstract

**Background:**

Pluripotency, the property of a cell to differentiate into all cellular types of a given organism, is central to the development of stem cell-based therapies and regenerative medicine. Stem cell pluripotency is the result of the orchestrated activation of a complex transcriptional network characterized by the expression of a set of transcription factors including the master regulators of pluripotency Nanog and Oct4. Recently, it has been shown that pluripotency can be induced in somatic cells by viral-mediated expression of the transcription factors Oct3/4, Sox2, Klf4, and c-Myc.

**Results:**

Here we show that 5-Aminoimidazole-4-carboxamide-1-b-riboside (AICAR) is able to activate the molecular circuitry of pluripotency in mouse embryonic stem cells (mESC) and maintain Nanog and Oct4 expression in mESC exposed to the differentiating agent retinoic acid. We also show that AICAR is able to induce Klf4, Klf2 and Myc expression in both mESC and murine fibroblasts.

**Conclusion:**

AICAR is able to activate the molecular circuitry of pluripotency in mESC and to induce the expression of several key regulators of pluripotency in somatic cells. AICAR is therefore a useful pharmacological entity for studying small molecule mediated induction of pluripotency.

## Background

Pluripotency, the ability to differentiate into all embryonic tissues, is a defining characteristic of embryonic stem cells and of induced pluripotent stem cells. Understanding how to induce, modulate, and maintain the pluripotent state of mammalian cells is of great importance for the development of critical tools for regenerative medicine. Pluripotency has been shown to be the product of an extended transcriptional network[1] that can be fully activated by the viral mediated overexpression of defined transcription factors: Klf4, Oct4, Sox2 and c-Myc[2-5]. Among these four factors, Klf4 has been suggested to have a higher functional hierarchical position [6] that is shared by other members of the Klf transcription factors family, including Klf2 [7].

While viral overexpression of transcription factors has been an invaluable tool to investigate the molecular basis of pluripotency, the presence in reprogrammed cells of viral genomes seems to be a barrier for the implementation of safe regenerative therapies. In light of this, small molecules are currently seen as plausible alternative to induce the expression of the critical transcription factors and, eventually, pluripotency [8-11].

Our laboratory has recently shown that the cell permeable nucleoside AICAR can induce the expression of different members of the Klf family of transcription factors in endothelial cells (H.B. Larman and G. Garcia-Cardena, unpublished observations). Thus, we hypothesized that AICAR could induce Klf2 and Klf4 expression in other cell types and that it could activate the pluripotency transcriptional network. Here we show that AICAR induces Klf2 and Klf4 expression and activates the pluripotency transcriptional network in mESC. This effect is able to antagonize mESC retinoic acid induced differentiation. Moreover, AICAR is able to induce Klf4, Klf2 and Myc expression in mouse embryonic fibroblasts, the prototypical somatic cells used for reprogramming studies. Our data describes a new property of AICAR in modulating mESC pluripotency network, and defines this small molecule as a new potential tool for the pharmacological reprogramming of somatic cells.

## Results

### AICAR induces the expression of Klf2 and Klf4 and activates the pluripotency transcriptional network in mESC

**Figure 1:**
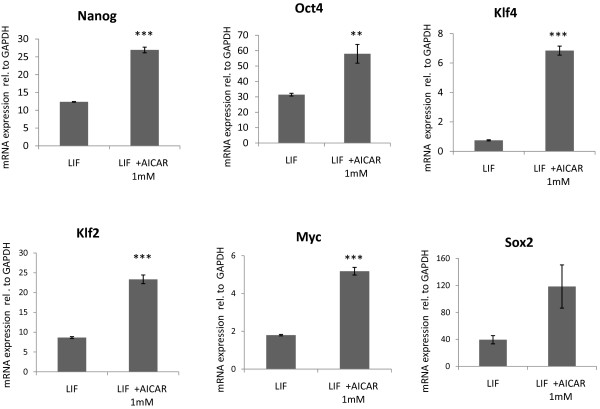
**AICAR activates the pluripotency transcriptional network in mESC**. Murine embryonic stem cells were exposed to 1 mM AICAR for 72 h. AICAR induced upregulation of the master regulators of pluripotency Nanog and Oct4 and of the pluripotency related transcription factors Klf4, Klf2, Myc and Sox2. Taqman real time quantitative PCR. N = 3. Graphs represent average +/- SEM. *** = P < 0.0001, ** = P < 0.001.

The J1 mESC line is a well characterized pluripotent cell line derived from the 129 Sv/J mouse strain. This cell line was used to assess the effects of AICAR on gene expression in pluripotent cells. ES cells were plated at 5000/cm^2 ^and exposed to 1 mM AICAR for 72 h. Gene expression was measured by total RNA extraction followed by RNA reverse transcription and real-time Taqman quantitative PCR. As seen in Figure 1, AICAR induced a 9 fold upregulation of Klf4 and a 2.7 fold upregulation of Klf2. Concomitantly, AICAR treatment induced the activation of the pluripotency transcription network as demonstrated by the upregulation of the pluripotency keeper transcription factors Nanog [12,13], Oct4 [14], Myc [15] and Sox2 [16]

### AICAR antagonizes Retinoic Acid induced differentiation of mESC

**Figure 2:**
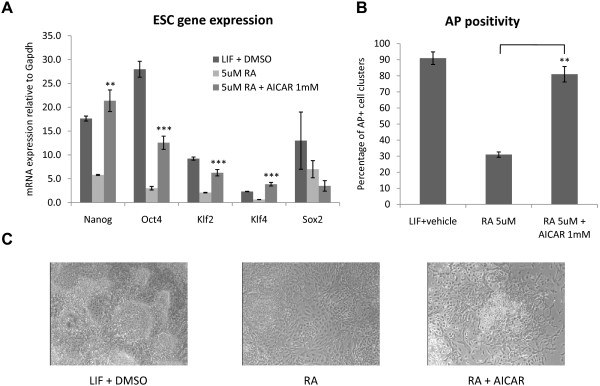
**AICAR antagonizes Retinoic Acid induced differentiation in mESC**. **A**) Murine embryonic stem cells were exposed to DMSO, 5 uM retinoic acid (RA) or 5 uM retinoic acid + 1 mM AICAR for 72 h. AICAR antagonized RA induced silencing of Nanog, Oct4, Klf2 and Klf4 but not of Sox2. Taqman real time quantitative PCR. N = 3. Graphs represent average +/- SEM **B**) mESC were grown for 5 days in the presence of DMSO, 5 uM RA or 5 uM RA + 1 mM AICAR and then stained for alkaline phosphatase activity. AICAR antagonizes RA induced suppression of AP activity. Representative experiment of 4 independent experiments that produced similar results. Bar represents average percentage of AP+ colonies per 4× magnification field +/- STDV. **C**) mESC were grown for 5 days in the presence of DMSO, 5 uM RA or 5 uM RA + 1 mM AICAR and then visually inspected for morphological analysis. AICAR reduces RA induced morphological changes. *** = P < 0.0001, ** = P < 0.001.

In order to further assess the effects of AICAR on the pluripotency transcriptional network, mESC were treated with AICAR in the presence of high doses of Retinoic Acid (RA), a well-characterized differentiating agent that induces downregulation of several members of the pluripotency transcriptional network, including Klf4, Klf2, Nanog, Oct4 and Sox2 [17]. As shown in Figure 2A, AICAR rescued RA mediated downregulation of Nanog (complete rescue), Oct4 (partial rescue), Klf4 (complete rescue) and Klf2 (partial rescue). AICAR didn't rescue RA mediated Sox2 downregulation. The analysis of the AICAR effects on RA induced differentiation by morphological and alkaline phosphatase criteria [17] confirmed the ability of AICAR to partially antagonize RA induced differentiation. In mESC colonies exposed to RA, AICAR was in fact able to significantly rescue the loss of alkaline phosphatase positivity associated with differentiation (Fig 2B) and, to a lesser extent, to reduce differentiation related colony morphological changes (Fig 2C). These results were confirmed with the E14 mESC line.

### AICAR induces Klf4, Klf2 and Myc upregulation in mouse embryonic fibroblasts

**Figure 3:**
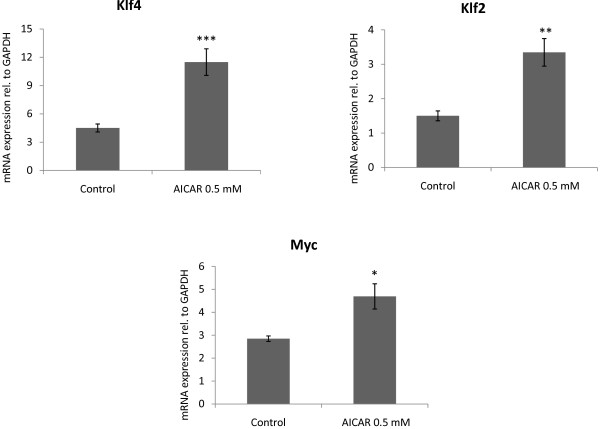
**AICAR induces Klf4, Klf2 and Myc expression in fibroblasts**. Murine embryonic fibroblasts were cultured in the presence or absence of AICAR for 48 h. AICAR induced upregulation of Klf4, Klf2 and Myc. Taqman real time quantitative PCR. N = 3. Graphs represent average +/- SEM. *** = P < 0.0001, ** = P < 0.001, * = P < 0.05.

Embryonic fibroblasts are the prototypical cells used to study transcription factor induced somatic reprogramming [2,18,19]. We therefore tested the effect of AICAR on mouse embryonic fibroblasts (MEFs). As shown in Figure 3, AICAR induces expression of Klf4 (2.6 fold), Klf2 (2.2 fold) and Myc (1.7 fold) in MEFs. However, under these conditions, AICAR was not able to orchestrate the complete activation of the transcriptional pluripotency network as documented by the absence of AICAR-induced expression of Nanog, Oct4 or Sox2 (data not shown).

## Discussion

Pluripotency is the product of the activation of a complex transcriptional network that induces expression of genes encoding transcription factors, signal transduction components, and chromatin-modifying enzymes. The pluripotency transcriptional network seems to be the same in both embryonic stem cells and induced pluripotent stem cells (somatic cells reprogrammed to gain pluripotency). While there have been few reports of small molecules that can maintain pluripotency of embryonic stem cells [20-22], there are no reports of molecules tested for their ability of both modulating the transcriptional network in embryonic stem cells and regulating the expression of pluripotency inducing transcription factors in somatic cells.

In the present work we show that AICAR is able to activate the pluripotency transcriptional network in mESC and to upregulate Klf4, Klf2 and Myc expression in murine fibroblasts. The effect of AICAR as activator of the pluripotency molecular circuitry is clearly shown by the AICAR-mediated induction of the master regulators of pluripotency Nanog, Oct4, Myc and Sox2 and by the AICAR-mediated induction of Klf4 and Klf2, two transcription factors highly expressed in undifferentiated ESCs, which are quickly downregulated upon induction of differentiation[17]. The effect of upregulating these transcription factors on the pluripotency transcriptional network is further supported by the ability of AICAR to antagonize RA-induced differentiation. While the AICAR effect is not dominant over the differentiating action of RA, both the gene expression analysis, and the alkaline phosphatase staining clearly demonstrate that AICAR significantly antagonizes RA induced differentiation by competing against RA mediated silencing of pluripotency related genes.

Our experiments with MEFs show that AICAR is able to upregulate Klf4, Klf2 and Myc also in somatic cells. In contrast to the effects exerted on mESC, in this context AICAR is not able to induce Oct4, Nanog or Sox2 expression and is therefore not able to activate the pluripotency network. This result is not surprising if we consider that while Klf4 has been suggested to be able to regulate the expression of all the transcription factors required for somatic cell reprogramming [6], this ability is conditional to a proper chromatin status and the function of a set of coactivators that are likely not present in fibroblasts in the absence of other stimuli. Moreover, AICAR induces a Klf4 upregulation of about 2.5 fold. This upregulation is much smaller than the Klf4 upregulation obtained by the viral mediated overexpression methods used in all the somatic cell reprogramming protocols described [2]. While AICAR by itself is not able to reprogram fibroblasts, AICAR mediated Klf4 induction in somatic cells is of particular interest in the context of the current quest for small molecule mediated induction of pluripotency. Moreover, the fact that AICAR promotes pluripotency in mESC suggests that AICAR effect on Klf4 transcription is not linked to other pro differentiating actions and that AICAR might be successfully used in pluripotency inducing small molecules cocktails.

AICAR has been previously shown to have a number of effects on cellular function [23-31] including effects on cellular differentiation. AICAR has been in fact shown to have anti-differentiating effects (in adypocytes [32] and myoblasts [33]) and pro-differentiating effects (in neural stem cells [34] and endothelial progenitor cells [35]). We now report an effect of AICAR as antagonist of retinoic acid induced differentiation in embryonic stem cells. The molecular basis of these contrasting effects of AICAR on cell differentiation is not clear but it might reflect the fact that different molecular mechanisms maintain different levels of multipotency in different cell types[36].

Our present data clearly do not provide a mechanism underlying AICAR effects on mESC pluripotency and Klf transcription in fibroblasts. Since AICAR has been previously shown to prevent skeletal myoblasts differentiation via activation of the longevity regulator Sirt1 [33], it is intriguing to speculate that Sirt1 might be downstream of AICAR in the regulation of embryonic stem cell pluripotency, establishing a connection between the molecular mechanism of aging and the molecular basis of pluripotency. AICAR mediated upregulation of Klf4 and Klf2 in MEFs could be mediated by the same mechanism underlying activation of the pluripotency network in mESC or by a different one, like activation of MEF2, a known target of AICAR in muscle cells [37] and a known regulator of Klf4 and Klf2 expression in endothelial cells[38]

## Conclusion

Collectively, this work describes for the first time a small molecule that is both able to activate the pluripotency transcriptional network in mESC, and to induce the expression in somatic cells of two of the four transcription factors required for reprogramming. Our study identifies AICAR as a pharmacological space important for the establishment and/or maintenance of induced pluripotent stem cells.

## Methods

### Cell culture

The J1 mouse embryonic stem cell line (a gift of Dr. George Daley, Children's Hospital, Boston) was gelatin adapted and grown on 1% gelatin coated tissue culture flasks in DMEM (Invitrogen, USA) 10% FCS, 1× non essential amino acids (Invitrogen, USA), 55 μM β-mercaptoethanol (Invitrogen, USA), Penicillin 10 units/ml (Invitrogen, USA), Streptomycin 10 μg/ml (Invitrogen, USA), 1000 U/ml LIF (ESGRO, Millipore, USA) and split by trypsinization every 2–3 days. The E14 mouse embryonic stem cell (gift of Dr George Daley, Children's Hospital, Boston) line was cultured in similar conditions but on a feeder layer of mytomycin-treated murine embryonic fibroblasts (Millipore, USA). E14 mESC were deprived of contaminating embryonic fibroblasts by trypsinization and 45 minutes incubation on non-gelatinized tissue culture flasks prior to plating for exposure to AICAR.

Strain CF-1 Mouse embryonic fibroblasts were purchases from Millipore (USA) and cultured in DMEM (Invitrogen, USA) 10% FCS, Penicillin 10 units/ml (Invitrogen, USA), Streptomycin 10 ug/ml (Invitrogen, USA).

### Gene expression analysis

Total RNA was extracted with the ABI 6100 Nucleic Acid PreStation (Applied Biosystems, Foster City, CA) according to manufacturer's instructions. Reverse transcription of RNA was performed with Multiscribe reverse transcriptase (Applied Biosystems, Foster City, CA) according to manufacturer's instruction. Real-time Taqman PCR was performed in 20 μl reactions with primers from Applied Biosystems, according to manufacturer's instructions.

### Exposure to AICAR and Retinoic Acid induced differentiation

5-Aminoimidazole-4-carboxamide-1-β-riboside (Calbiochem, San Diego, CA) was dissolved in PBS without calcium at a 100 mM final concentration. All trans-retinoic acid (Sigma, St Louis, MO) was dissolved in DMSO at a final concentration of 2.5 mM. To induce mESC differentiation, Retinoic Acid was added to mESC culture media without LIF at a final concentration of 5 micromoles per liter. Media with RA and or AICAR was replaced every 24 hours.

### Alkaline phosphatase staining

Growing cells were washed in PBS, fixed for one minute in 4% PFA PBS, and then incubated for 30 minutes in BM purple alkaline phosphatase (AP) staining solution (Roche applied sciences, USA). The percentage of alkaline phosphatase positive colonies was calculated counting the number of AP+ and AP- cell clusters in three different fields at a 4× magnification.

### Statistical Analysis

Two way analysis was performed with un-paired t-test assuming samples with equal variance. Three way analysis was performed with single factor ANOVA.

## Authors' contributions

LA and GGC designed the experiments. LA and YZ performed the experiments. LA wrote the manuscript under the supervision of GGC.

## References

[B1] Wang J, Rao S, Chu J, Shen X, Levasseur DN, Theunissen TW, Orkin SH (2006). A protein interaction network for pluripotency of embryonic stem cells. Nature.

[B2] Takahashi K, Yamanaka S (2006). Induction of pluripotent stem cells from mouse embryonic and adult fibroblast cultures by defined factors. Cell.

[B3] Takahashi K, Tanabe K, Ohnuki M, Narita M, Ichisaka T, Tomoda K, Yamanaka S (2007). Induction of pluripotent stem cells from adult human fibroblasts by defined factors. Cell.

[B4] Wernig M, Zhao JP, Pruszak J, Hedlund E, Fu D, Soldner F, Broccoli V, Constantine-Paton M, Isacson O, Jaenisch R (2008). Neurons derived from reprogrammed fibroblasts functionally integrate into the fetal brain and improve symptoms of rats with Parkinson's disease. Proc Natl Acad Sci USA.

[B5] Hanna J, Markoulaki S, Schorderet P, Carey BW, Beard C, Wernig M, Creyghton MP, Steine EJ, Cassady JP, Foreman R (2008). Direct reprogramming of terminally differentiated mature B lymphocytes to pluripotency. Cell.

[B6] Kim J, Chu J, Shen X, Wang J, Orkin SH (2008). An extended transcriptional network for pluripotency of embryonic stem cells. Cell.

[B7] Jiang J, Chan YS, Loh YH, Cai J, Tong GQ, Lim CA, Robson P, Zhong S, Ng HH (2008). A core Klf circuitry regulates self-renewal of embryonic stem cells. Nat Cell Biol.

[B8] Meissner A, Wernig M, Jaenisch R (2007). Direct reprogramming of genetically unmodified fibroblasts into pluripotent stem cells. Nat Biotechnol.

[B9] Gearhart J, Pashos EE, Prasad MK (2007). Pluripotency redux – advances in stem-cell research. N Engl J Med.

[B10] Holden C (2007). Stem cells. Teams reprogram differentiated cells – without eggs. Science.

[B11] Huangfu D, Osafune K, Maehr R, Guo W, Eijkelenboom A, Chen S, Muhlestein W, Melton DA (2008). Induction of pluripotent stem cells from primary human fibroblasts with only Oct4 and Sox2. Nat Biotechnol.

[B12] Chambers I, Colby D, Robertson M, Nichols J, Lee S, Tweedie S, Smith A (2003). Functional expression cloning of Nanog, a pluripotency sustaining factor in embryonic stem cells. Cell.

[B13] Chambers I, Silva J, Colby D, Nichols J, Nijmeijer B, Robertson M, Vrana J, Jones K, Grotewold L, Smith A (2007). Nanog safeguards pluripotency and mediates germline development. Nature.

[B14] Nichols J, Zevnik B, Anastassiadis K, Niwa H, Klewe-Nebenius D, Chambers I, Scholer H, Smith A (1998). Formation of pluripotent stem cells in the mammalian embryo depends on the POU transcription factor Oct4. Cell.

[B15] Cartwright P, McLean C, Sheppard A, Rivett D, Jones K, Dalton S (2005). LIF/STAT3 controls ES cell self-renewal and pluripotency by a Myc-dependent mechanism. Development.

[B16] Avilion AA, Nicolis SK, Pevny LH, Perez L, Vivian N, Lovell-Badge R (2003). Multipotent cell lineages in early mouse development depend on SOX2 function. Genes Dev.

[B17] Ivanova N, Dobrin R, Lu R, Kotenko I, Levorse J, DeCoste C, Schafer X, Lun Y, Lemischka IR (2006). Dissecting self-renewal in stem cells with RNA interference. Nature.

[B18] Schenke-Layland K, Rhodes KE, Angelis E, Butylkova Y, Heydarkhan-Hagvall S, Gekas C, Zhang R, Goldhaber JI, Mikkola HK, Plath K (2008). Reprogrammed Mouse Fibroblasts Differentiate into Cells of the Cardiovascular and Hematopoietic Lineages. Stem Cells.

[B19] Mikkelsen TS, Hanna J, Zhang X, Ku M, Wernig M, Schorderet P, Bernstein BE, Jaenisch R, Lander ES, Meissner A (2008). Dissecting direct reprogramming through integrative genomic analysis. Nature.

[B20] Chen S, Do JT, Zhang Q, Yao S, Yan F, Peters EC, Scholer HR, Schultz PG, Ding S (2006). Self-renewal of embryonic stem cells by a small molecule. Proc Natl Acad Sci USA.

[B21] Miyabayashi T, Teo JL, Yamamoto M, McMillan M, Nguyen C, Kahn M (2007). Wnt/beta-catenin/CBP signaling maintains long-term murine embryonic stem cell pluripotency. Proc Natl Acad Sci USA.

[B22] Chen L, Khillan JS (2008). Promotion of Feeder Independent Self-Renewal of Embryonic Stem Cells By Retinol (Vitamin A). Stem Cells.

[B23] Merrill GF, Kurth EJ, Hardie DG, Winder WW (1997). AICA riboside increases AMP-activated protein kinase, fatty acid oxidation, and glucose uptake in rat muscle. Am J Physiol.

[B24] Musi N, Hayashi T, Fujii N, Hirshman MF, Witters LA, Goodyear LJ (2001). AMP-activated protein kinase activity and glucose uptake in rat skeletal muscle. Am J Physiol Endocrinol Metab.

[B25] Muoio DM, Seefeld K, Witters LA, Coleman RA (1999). AMP-activated kinase reciprocally regulates triacylglycerol synthesis and fatty acid oxidation in liver and muscle: evidence that sn-glycerol-3-phosphate acyltransferase is a novel target. Biochem J.

[B26] Kaushik VK, Young ME, Dean DJ, Kurowski TG, Saha AK, Ruderman NB (2001). Regulation of fatty acid oxidation and glucose metabolism in rat soleus muscle: effects of AICAR. Am J Physiol Endocrinol Metab.

[B27] Zhang L, Li J, Young LH, Caplan MJ (2006). AMP-activated protein kinase regulates the assembly of epithelial tight junctions. Proc Natl Acad Sci USA.

[B28] Swinnen JV, Beckers A, Brusselmans K, Organe S, Segers J, Timmermans L, Vanderhoydonc F, Deboel L, Derua R, Waelkens E (2005). Mimicry of a cellular low energy status blocks tumor cell anabolism and suppresses the malignant phenotype. Cancer Res.

[B29] Rencurel F, Foretz M, Kaufmann MR, Stroka D, Looser R, Leclerc I, da Silva Xavier G, Rutter GA, Viollet B, Meyer UA (2006). Stimulation of AMP-activated protein kinase is essential for the induction of drug metabolizing enzymes by phenobarbital in human and mouse liver. Mol Pharmacol.

[B30] Lee K, Li B, Xi X, Suh Y, Martin RJ (2005). Role of neuronal energy status in the regulation of adenosine 5'-monophosphate-activated protein kinase, orexigenic neuropeptides expression, and feeding behavior. Endocrinology.

[B31] McCullough LD, Zeng Z, Li H, Landree LE, McFadden J, Ronnett GV (2005). Pharmacological inhibition of AMP-activated protein kinase provides neuroprotection in stroke. J Biol Chem.

[B32] Giri S, Rattan R, Haq E, Khan M, Yasmin R, Won JS, Key L, Singh AK, Singh I (2006). AICAR inhibits adipocyte differentiation in 3T3L1 and restores metabolic alterations in diet-induced obesity mice model. Nutr Metab (Lond).

[B33] Fulco M, Cen Y, Zhao P, Hoffman EP, McBurney MW, Sauve AA, Sartorelli V (2008). Glucose restriction inhibits skeletal myoblast differentiation by activating SIRT1 through AMPK-mediated regulation of Nampt. Dev Cell.

[B34] Zang Y, Yu LF, Pang T, Fang LP, Feng X, Wen TQ, Nan FJ, Feng LY, Li J (2008). AICAR induces astroglial differentiation of neural stem cells via activating the JAK/STAT3 pathway independently of AMP-activated protein kinase. J Biol Chem.

[B35] Li X, Han Y, Pang W, Li C, Xie X, Shyy JY, Zhu Y (2008). AMP-activated protein kinase promotes the differentiation of endothelial progenitor cells. Arterioscler Thromb Vasc Biol.

[B36] Mikkers H, Frisen J (2005). Deconstructing stemness. Embo J.

[B37] Al-Khalili L, Chibalin AV, Yu M, Sjodin B, Nylen C, Zierath JR, Krook A (2004). MEF2 activation in differentiated primary human skeletal muscle cultures requires coordinated involvement of parallel pathways. Am J Physiol Cell Physiol.

[B38] Parmar KM, Larman HB, Dai G, Zhang Y, Wang ET, Moorthy SN, Kratz JR, Lin Z, Jain MK, Gimbrone MA (2006). Integration of flow-dependent endothelial phenotypes by Kruppel-like factor 2. J Clin Invest.

